# Supplementary material for: The genome sequence of the Dusky Meadow Brown, Cercyonis lycaon (Lepidoptera: Nymphalidae)
Source: Wellcome Open Res. 2025 Jul 31;10:395. [Version 1] doi: 10.12688/wellcomeopenres.24656.1 (PMC12411839; doi:10.12688/wellcomeopenres.24656.1)
Supplement: Peer review history

# Reviewer report: Reviewer response for version 1

**DOI:** 10.21956/wellcomeopenres.27170.r128202

**Published:** 2025-09-11

**Authors:** Arun Sethuraman, Jackson Wolfe

**Affiliations:** 1 Biology, San Diego State University College of Sciences (Ringgold ID: 115104), San Diego, California, USA; 2 San Diego State University, San Diego, California, USA

Summary: The authors report a high quality nuclear genome (~600Mb) of the dusky meadow brown, with 95% of the genome assembled into 30 chromosomal scaffolds, and a mitochondrial genome of 15.2 kb. 

 The authors do a good job of describing the methods used in generating the assembly. The introduction section included sufficient biological information regarding the rationale for the project.

 No major concerns were noted by the reviewers, all methods are accessible and reproducible, with the software and its parameters. The genome itself available and open for reuse via EBI.

Are sufficient details of methods and materials provided to allow replication by others?

Yes

Is the rationale for creating the dataset(s) clearly described?

Yes

Are the datasets clearly presented in a useable and accessible format?

Yes

Are the protocols appropriate and is the work technically sound?

Yes

Reviewer Expertise:

Genomics, population genetics, evolution

We confirm that we have read this submission and believe that we have an appropriate level of expertise to confirm that it is of an acceptable scientific standard.

# Reviewer report: Reviewer response for version 1

**DOI:** 10.21956/wellcomeopenres.27170.r128205

**Published:** 2025-09-04

**Authors:** Arun Arumugaperumal

**Affiliations:** 1 Department of Biotechnology, Rajalakshmi Engineering College, Thandalam 602105, Chennai, Tamil Nadu, 602105, India

The data note describes the genome sequence of *Cercyonis lycaon*, commonly known as the Dusky Meadow Brown. The photograph of the specimen remains in Figure 1 is of very good quality. This is the first report of the high quality genome of the butterfly species. The assembly reported is of size 601 Mb distributed among 30 chromosomes. The mitogenome has also been assembled of size 15.2 kb. The genomic DNA was sequenced using high-end sequencing machines. The assembly was said to be decontaminated by ASCC pipeline. What was the result? Does the presented genome size after removal of considerable amount of bases? The blob plot also shows emergence of a different blob.

 The links provided in the data note pointing to databases were working fine. A BUSCO completeness of 98.5% indicates that the genome is a near-complete one. The data note can be indexed.

Are sufficient details of methods and materials provided to allow replication by others?

Yes

Is the rationale for creating the dataset(s) clearly described?

Yes

Are the datasets clearly presented in a useable and accessible format?

Yes

Are the protocols appropriate and is the work technically sound?

Yes

Reviewer Expertise:

Bioinformatics; Genomics

I confirm that I have read this submission and believe that I have an appropriate level of expertise to confirm that it is of an acceptable scientific standard.

# Reviewer report: Reviewer response for version 1

**DOI:** 10.21956/wellcomeopenres.27170.r128203

**Published:** 2025-08-30

**Authors:** Annabel Whibley

**Affiliations:** 1 Grapevine Improvement, Bragato Research Institute, Lincoln, New Zealand; 2 The University of Auckland, Auckland, New Zealand

Chittaro and colleagues report the genome assembly of the Dusky Meadow Brown butterfly *(Cercyonis lyacon).  *This species was sequenced as part of Project Psyche, a multi-stakeholder project with an overarching goal to sequence all European Lepidopteran species.

 The background section sets out very effectively the natural history, population distribution, taxonomic context and karyotype evidence of *C. lyracon* and the voucher specimen photograph is of good quality. The sample processing methods section contains hyperlinks to protocols which have been given DOIs. I assume that a new DOI would be minted when the protocol version is updated, but I wonder whether it would be helpful to also note the protocol versions in the text itself.

 Combining Revio HiFi data and Arima Hi-C, the genome assembly is of exceptional quality (formally 6.C.Q64.). Close to 95% of scaffolds have been assigned to chromosomes, and BUSCO and kmer completeness are >98% and >99% respectively. formally 6.C.Q64. The use of a female individual means that both Z and W chromosomes have been captured, which adds to the utility of the reference. The inclusion of analyses of Merian elements, reconstructing ancestral linkage groups, is a really nice addition to standard elements of the DToL reporting. As usual for the data notes, the report follows templating and the methodology, reporting of sample metadata, protocols and assembly protocols are comprehensively and clearly documented.

 Please note there is a typo in Table 4 (for the EBP summary of haplotype 1 Value; it should be 6.C.Q64 not 6.7.Q64).

Are sufficient details of methods and materials provided to allow replication by others?

Yes

Is the rationale for creating the dataset(s) clearly described?

Yes

Are the datasets clearly presented in a useable and accessible format?

Yes

Are the protocols appropriate and is the work technically sound?

Yes

Reviewer Expertise:

Genomics, Bioinformatics

I confirm that I have read this submission and believe that I have an appropriate level of expertise to confirm that it is of an acceptable scientific standard.
